# Rethinking max–min planning on energy-efficient software-defined networking for 5G networks

**DOI:** 10.1038/s41598-024-76504-9

**Published:** 2024-10-28

**Authors:** Dingde Jiang, Bowen Zhu, Junyang Sun, Zhihao Wang, Zhihan Lyu, Amit Kumar Singh, Zhen Yuan

**Affiliations:** 1 School of Information and Communication Engineering, University of Electronic Science and Technology of China, Chengdu, 611731 China; 2 School of Information, North China University of Technology, Beijing, 100144 China; 3 School of Computer Science and Engineering, Northeastern University, Shenyang, 110819 China; 4 Faculty of Arts, Department of Game Design, Uppsala University, Uppsala, Sweden; 5 Department of Computer Science and Engineering, National Institute of Technology Patna, Patna, 800005 India

**Keywords:** Energy efficiency networks, 5G networks, Max–min planning, Software-defined networking, Intelligent networking, Electrical and electronic engineering, Computer science, Information technology, Information theory and computation

## Abstract

Energy efficiency plays an important role in intelligent networking for 5G networks, which concerns environmental, financial, and performance aspects of intelligent networking for 5G networks. To this end, network designers propose energy-efficient approaches to reduce energy consumption of networks and to raise network performance by switching off the links/nodes with low loads or at idle status. The existing energy-efficient approaches can be formulated as a max–min optimal problem, namely maximizing network/node/port throughput via minimum energy consumption. The max–min planning investigates energy efficiency only from the links/nodes perspective. The max–min planning for energy-efficient networking, if not carefully designed from the network-wide standpoint, can lead to lower energy efficiency for the whole network due to lack of global planning, which in turn results in the degraded performance due to network un-connectivity after closing the nodes/links. In this paper we rethink the max–min planning framework on energy-efficient software-defined networking for intelligent networking of 5G networks, which takes in account combining network connectivity and maximum network flow with minimum energy consumption. Our framework aims at how to deliver dynamic end-to-end traffic demands with the appropriate network topology by building data forwarding plane with maximum network flow and control plane with network connectivity. We discuss the associated challenges and implementation issues. A dynamic max–min planning framework depending on dynamic end-to-end traffic demands is presented to achieve network-wide energy efficiency. Numerical results show the improved energy efficiency performance for the whole network.

## Introduction

Continuous advancement in data traffic and network applications has resulted in high energy consumption for 5G networks. To transfer every gigabyte (GB) of data, the energy consumption of 4G is 0.117kWh, while it is 0.501kWh in 5G^1^. At the same time, the information and communications technology (ICT) industry consume up to 7% of global electricity^1^. The concerns have motivated research in energy-efficient planning for intelligent networking for 5G networks based on Software-Defined Networking (SDN)^2–4^. Energy efficiency plays an important role in 5G networks, which concerns environmental, financial, and performance aspects of intelligent networking for 5G networks^5^. Some energy-efficient approaches are proposed to reduce energy consumption of networks and to raise network performance by switching off the links/nodes with low loads or at idle status. Traffic-aware and energy-aware approaches are employed to achieve the energy-efficient networking by seeking the max–min optimal planning solution. Resource optimization^6^, relay selection^7^, and packet processing engine optimization^8^ are also used to raise energy efficiency. In general, the existing energy-efficient approaches can be formulated as a max–min optimal problem, namely maximizing network/node/port throughput via minimum energy consumption.

Besides, consideration of end-to-end traffic dynamics also plays an important role in the design and optimization of energy-efficient networks. Periods of reduced end-to-end traffic can be leveraged to activate sleep modes in network elements. Energy-efficient 5G networks intelligently manage power states, allowing certain components to enter low-power modes during lulls in activity^9^. This strategic use of sleep modes contributes to substantial energy savings without compromising responsiveness. Knowledge of end-to-end traffic dynamics informs the integration of green networking technologies^10^. Energy-efficient hardware, renewable energy sources, and innovative cooling systems are strategically employed based on traffic patterns, contributing to a more sustainable and environmentally friendly 5G network operation^5^.

To this end, when nodes/links carry the low traffic load, deactivation strategies are proposed to switch them off and to redirect the traffic on them to other the under-utilized links/nodes. If staying at a idle status, they are directly turned off. One limitation of the max–min planning is that it investigates energy efficiency only from the links/nodes perspective. The max–min planning for energy-efficient networking, if not carefully designed from the network-wide standpoint, can lead to lower energy efficiency for the whole network due to lack of global planning.

In such a context, the network-wide maximum flow delivery is difficult to be achieved to follow end-to-end traffic demands. Moreover, this results in the degraded performance due to network un-connectivity after closing the nodes/links. Additionally, 5G networks expect highly reachable, connective, real-time requirements^11–14^. Hence, the max–min planning should be redesigned to achieve energy-efficient networking.

In this paper, we rethink the max–min planning framework on energy-efficient SDN for intelligent networking of 5G networks to motivate research on this topic, which takes into account combining network connectivity and maximum network flow with minimum energy consumption. Our framework aims at how to deliver dynamic end-to-end traffic demands with the appropriate network topology by building data forwarding plane with maximum network flow and control plane with network connectivity in SDN. Firstly, the fundamentals of the existing max–min planning approaches are reviewed. Then a dynamic max–min planning framework depending on dynamic end-to-end traffic demands is proposed and the associated challenging design and implementation issues are discussed. Finally, a dynamic max–min planning approach for energy efficiency is presented, and numerical results are proposed to demonstrate the performance of our approach.

## Max–min planning in energy-efficient networks

In this section we formulate the motivation and design fundamentals for max–min planning for intelligent networking of 5G networks using SDN .

### Motivations of max–min planning

In current communication network planning, the redundant paths are designed to guarantee the network reliability. However, the single path of packet deliveries makes it significantly difficult to meet end-to-end users’ traffic demands for 5G networks. One solution is to provide the redundant link capacity. Although the redundant design of link capacities is used to follow the peak traffic demand, the over-provisioned design leads to higher energy consumption along with a lower traffic load. Another possible solution is mesh network design. Mesh network design is too expensive to practice due to deploy overhead and high-performance devices, specially for 5G networks. Consequently, the maximum network flow delivery strategy from source nodes to destination nodes is always used to conduct network designs that are satisfying with maximum end-to-end traffic demands as possible. The maximum network flow attains end-to-end packet delivery performance from the network-wide perspective. Thus, this possibly makes more links take part in end-to-end packet forwarding. The whole network yields high energy consumption. Thus the energy-efficient networking approach is proposed to implement the largest network flow forwarding while expending the low energy for 5G networks.

In such a case, two fundamental problems must be handled while designing energy-efficient networks for 5G networks, namely maximum network flows and minimum network energy consumption.

### Maximum network flows

In energy-efficient networks, maximum flow deliveries must be considered to raise network throughput. This issue deals with how to maximize network flow to attain maximum network throughput. In the following, we discuss the design objectives of the maximum network flow mechanism in network planning for 5G networks. Then we review the maximum network flow mechanism decision criteria.

*Design Objectives:* Maximum network flow follows two design objectives for energy-efficient networks. The first objective is to maximize end-to-end network throughput to optimize network forwarding efficiency. The second objective aims to avoid end-to-end network congestion. The fundamental goal of these design objectives is to meet maximum end-to-end users’ traffic demands.

*Design Criteria:* The maximum link utilization is the first design criteria, where each link is sufficiently used according to its capacity constraints. The second design criteria is that the end-to-end traffic demands are balanced on each end-to-end path, which aims to avoid network congestion and packet loss. Moreover, maximizing the throughput of the whole network is another design criteria where the network flow deliveries between multiple source nodes and multiple destination nodes are maximized together.

### Minimum network energy consumption

The minimum network energy consumption specifies which links are turn off, when to wake up a given link, and how to implement the switching decisions. The associated design issues are discussed in the following.

*Prediction of End-to-End Traffic Demands:*The link traffic loads have a direct impact on link energy consumption^15^. The active/sleeping mode of links should dynamically change according to end-to-end flow traffic demands. The appropriate lasting duration in the active/sleeping mode is considered to avoid frequent link mode switching. Hence, the link mode active/sleeping switching decision should consider not only the current traffic load but also future demands so as to guarantee an acceptable Quality of Service (QoS) and Quality of Experience (QoE)^3,11,13,16^. As a result, extra resources should be reserved for the active links and paths to satisfy future end-to-end traffic demands^4^. End-to-end traffic estimation and prediction approaches can be used to infer the end-to-end traffic demand matrix^15^.

*Link Sleeping Design:*When the end-to-end traffic demands served by the active links increase beyond their capacity limitation, some of the switched-off links have to be turned on. Hence, in the max–min planning, it is necessary to specify the wake-up instants for the inactive links. The sleeping mechanism proposed^4,16^ or the defined sleeping time can be used to let links enter the sleeping status.

*Switching of Link Active/Sleeping Mode:* An end-to-end flow may not be able to complete its handoff procedure to an active path and suffer from packet loss if its associated links are switched off too quickly. This is due to lack of link synchronization and collaboration. The fixed wake-up time or the wake-up mechanism may be exploited to achieve the goal.

## Energy-efficient networking with max–min planning

The main goal of max–min planning is to reduce network energy consumption while maximizing end-to-end network throughput at any time. However, no attention is paid to the relation among maximum network flows, dynamic changes of end-to-end traffic demands, and their incurred network energy consumption. For instance, if an end-to-end traffic demand is low during a certain interval, it is expected to under-utilize the end-to-end path capacity, eventually leading the low link utilization while consuming large energy at a fast rate. Hence, the energy-efficient networking with dynamic max–min planning among end-to-end traffic demands and energy consumption should be investigated. In the following, the motivation for the dynamic max–min planning for energy-efficient networking is presented. Then challenging design and implementation issues are discussed.

**Fig. 1:**
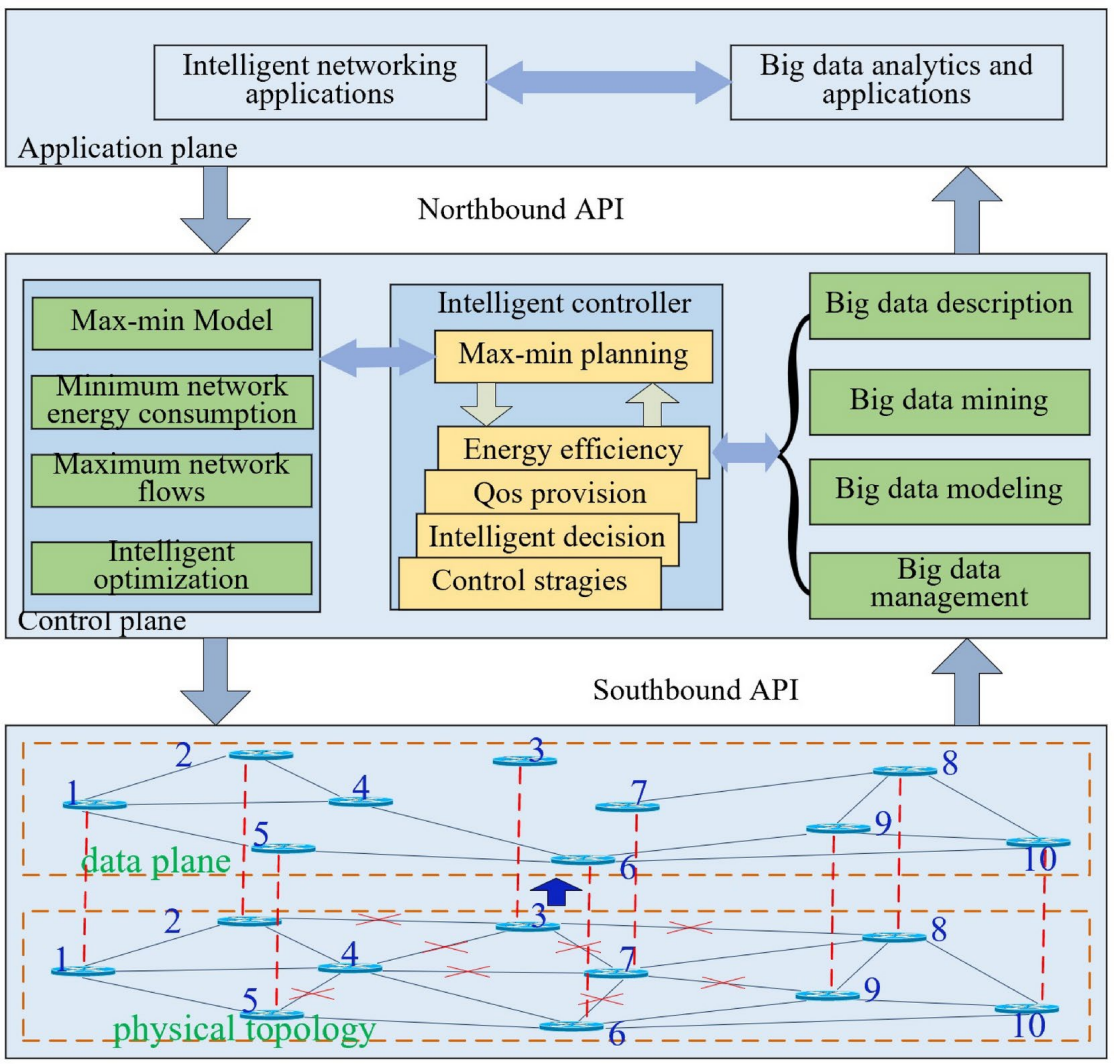
Max–min planning for energy efficiency with network-wide perspective based on SDN, with red notation ’x’ denoting low load or idle links. Node 3 is unreachable.

### Motivation

When the dynamic max–min planning is to enhance the energy efficiency of networks, the current active/sleeping switch-off decisions can result in low energy efficiency from the network-wide standpoint. As shown in Fig. 1, accounting only for network/links energy consumption or maximum network throughput, time-varying end-to-end traffic demands can be associated with low link/path utilization, due to a given network planning, e.g. link $l_{78}$. Moreover, there exist some certain unreachable node, e.g. node 3. Because of the lower utilization and relative high energy consumption, low energy efficiency of networks from the network-wide perspective is expected, which leads to low packet delivery efficiency and high energy draining. Although energy consumption for the under-utilized links/paths is not that much compared with that of the highly utilized ones, a large energy demand for networks still results in a high operation cost of services in the under-utilized links. Hence, the dynamic max–min planning approach should be designed to handle the problem depending on time-varying end-to-end traffic demands.

### Design challenges

In this subsection we discuss the challenging design and implementation issues toward developing a max–min planning approach with dynamic traffic demands for energy-efficient networking for 5G networks.

*Tradeoff between Maximum Network Flow Deliveries and Minimum Network Energy Consumptions:* One challenge with max–min planning is that the switching decisions of links are coupled with links’ utilization. Specifically, when a link is switched off, the paths associated with it need to perform a handover process to other links. Similarly, when a link is turned on, the paths can perform a handover process to the link for maximizing energy efficiency. Hence, depending on dynamic end-to-end traffic demands, the max–min planning decision is a dynamic process. Additionally, end-to-end traffic demands hold burst nature. Another challenge is how to meet the requirement.

**Fig. 2:**
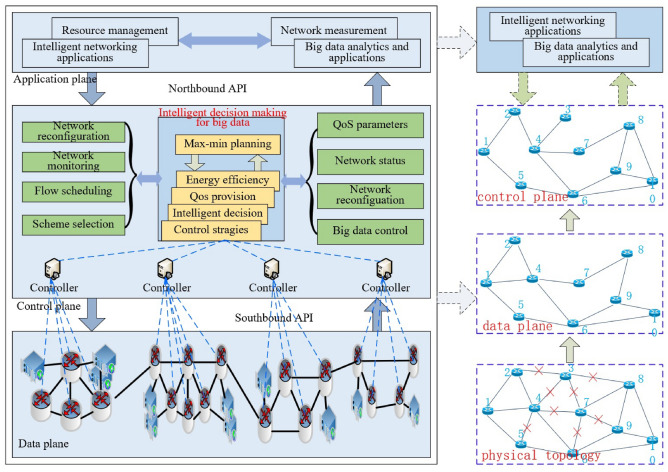
Max–min planning for energy efficiency with network-wide perspective for multiple controllers, with red notation ’x’ denoting low load or idle links. Network is divided into data plane and control plane, with the former responsible for data forwarding and with the latter responsible for network connectivity.

*New Design Paradigm:*When the time-varying nature of end-to-end traffic demands is considered for 5G networks, the link switch-off decision criteria should be revised. Specifically, the switch-off decision criteria should capture the impact of the dynamic changes of end-to-end traffic on network service performance, for example, lower throughput, higher energy consumption, higher delay, and so forth. The lower throughput and higher energy consumption are due to lack of network-wide planning, while the higher delay is because of the link switching or node unreachability. The end-to-end traffic demands exhibit slower change properties than link loads, while holding the definite periodicity nature (such as daily periodicity)^17^. Hence, the end-to-end traffic demands are divided in different time slots per day. In each time slots, the end-to-end traffic demand is used by control plane to calculate the energy-efficient forwarding paths. The first and most important issue to consider when calculating a forwarding path is end-to-end connectivity, which means there must be at least one path between the source node and the destination node. The calculated results should attain as much network flow as possible and minimize energy consumption, as detailed in the following sections. The calculated paths form a subset of the original network topology, including all switches and partially activated links, as shown in the topology of the data plane in Fig. 2. The remaining links do not forward traffic to save energy, as shown in the physical topology in Fig. 2. The paths are then distributed to the switches in the data plane, with northbound interfaces as flow table entries. The SDN switches in data plane forward the corresponding network flows by entry matching^18,19^. The switch only interacts with the controller when it first encounters the corresponding flow, which reduces the delay during forwarding traffic.

## Dynamic max–min energy-efficient approach for 5G networks intelligent networking

In this section we propose an energy-efficient approach with dynamic max–min planning for intelligent networking of 5G networks.

### System model

The related 5G architecture used in this model is based on Heterogeneous Cloud Radio Access Network (H-CRAN) that combines the advantages of Heterogeneous Network (HetNet) and Cloud Radio Access Network (C-RAN). HetNet separates the control and data plane using High Power Node (HPN). C-RAN utilizes Remote Radio Head (RRH) to efficiently support local businesses. Different from C-RAN, the Base Band processing Unit (BBU) pool in H-CRAN connects to the existing HPN, which allows for the full utilization of macro base stations in cellular networks such as 3G and 4G to achieve seamless coverage and separation of control and service plane functions. HPN is used for controlling information distribution across the entire network, separating the centralized control cloud function module from the BBU pool. In H-CRAN, all control signals and system broadcast information are sent by HPN to User Equipment (UE), which enables RRH to adaptively sleep according to user business needs, effectively saving energy consumption and achieving user centered green and energy-saving communication^20^. Based on the architecture of H-CRAN, we introduce the intelligent networking structure shown in Fig. 2. The separation of the control plane and data plane in H-CRAN allows us to use the control plane to generate the max–min planning and distribute the energy-efficient planning scheme to the data plane, which determines the on/off state of links. The structure in Fig. 2 is illustrated with multiple geographically distributed networks. Each sub-network is controlled by a distributed SDN controller that is aware of the state of the data plane and responsible for distributing the forwarding rules. All distributed controllers receive the global intelligent decision-making module that generates the max–min planning scheme. The states of the distributed controllers synchronize through distributed consensus algorithms. Let *n* denote the node index, with $n\in {1, 2,\ldots , 10}$. The end-to-end traffic demands are divided into $\pi \in {1, 2,\ldots , z}$ time slots according to traffic nature in a day, where the size of each time slot is not equal due to different properties in each hour per day, and denotes the number of divided time slots. In each time slot, the physical topology in Fig. 2 is exploited to build data plane and control plane, respectively, subjective to minimum energy consumption. The data plane in each time slot achieves maximum network flow delivery matching the end-to-end traffic demands, that is, the data plane delivers data packets via maximum link utilization while the paths built by maximum network flow method can exactly meet the end-to-end traffic demands. The control plane in each time slot guarantees network connectivity and is used for controlling network, such as link activation and sleeping in each time slot. Hence, for the network shown in Fig. 2, there exist $\pi$ data planes and control planes in a day, respectively. In each time slot, all data planes and control planes consume the minimum amount of energy from a network-wide perspective. In such a case, the minimum energy consumption is obtained to forward the maximum end-to-end traffic demands in each time slot.

### Max–min design formulation

The time in a day is divided into different time slots. In each time slot, there is a corresponding data plane and control plane. In each time slot, the end-to-end traffic demand, physical topology, maximum network flow, and minimum energy consumption are all considered to build the decision process. The process aims at establishing the optimal data plane and control plane with minimum energy consumption for matching end-to-end traffic demands.

**Algorithm 1:**
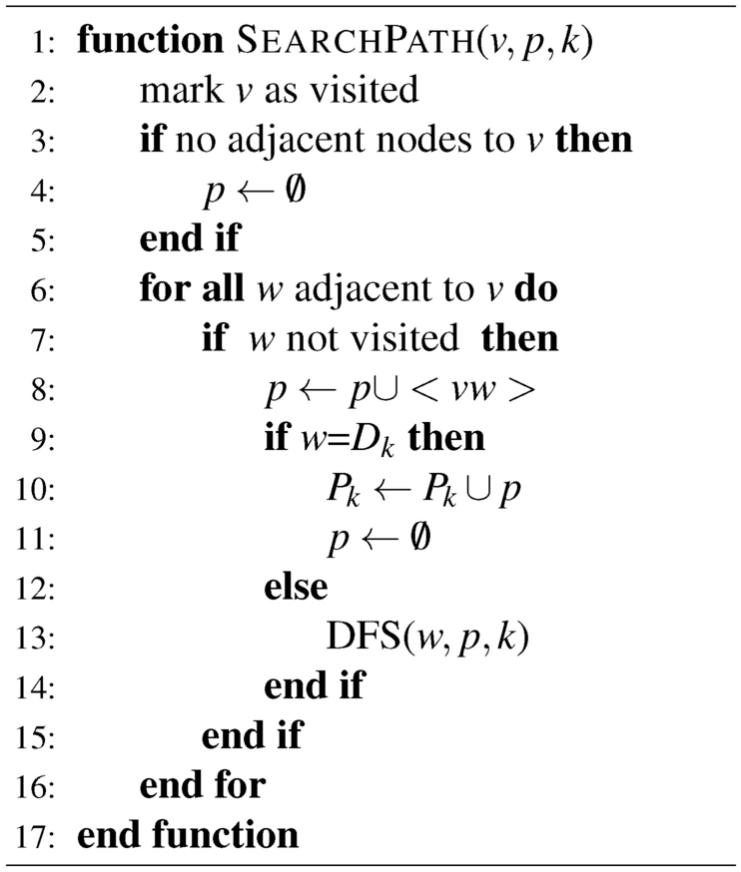
Deep First Path Searching Algorithm

*Maximum Network Flows:* Given a network $G = (V, E)$, the capacity of edge $<v_1 v_2>$ is $C_{v_1 v_2}$. For function $f: E \rightarrow R$ defined on *E*, if *f* meets capacity constraints (namely $\forall <v_1 v_2>$, $0 \le f_{v_1 v_2} \le C_{v_1 v_2}$) and flow conservation (namely ${\forall {v_i}} \in {V^{'}}$, $f^{in}(v_i) = {f^{out}}({v_i})$, where $V^{'} = V - (S,D)$), then *f* is referred to as a feasible flow on the data plane of *G* and $f_{v_1 v_2}$ is called the traffic of edge $<v_1 v_2>$, where *S* is the source node and *D* is the destination node. According to flow constraints of the data plane of *G*, the ingress and egress flow traffic of source node *S* and destination node *D* are, respectively, zero. The flow traffic conservation constraints of source nodes are formulated as follows:

1 $$\begin{aligned} \sum \limits _{{v_j} \in V} {{f_{{v_i}{v_j}}}} - \sum \limits _{{v_j} \in V} {{f_{{v_j}{v_i}}}} = {v_f} \quad {v_i} \in S,\ \left\langle {{v_i}{v_j}} \right\rangle \in E \end{aligned}$$

Similarly, flow traffic conservation constraints for destination nodes are formulated as follows:

2 $$\begin{aligned} \sum \limits _{{v_j} \in V} {{f_{{v_i}{v_j}}}} - \sum \limits _{{v_j} \in V} {{f_{{v_j}{v_i}}}} = - {v_f} \quad {v_i} \in D,\ \left\langle {{v_i}{v_j}} \right\rangle \in E \end{aligned}$$

The flow traffic conservation constraints of intermediate nodes are formulated as:

3 $$\begin{aligned} \sum \limits _{{v_j} \in V} {{f_{{v_i}{v_j}}}} - \sum \limits _{{v_j} \in V} {{f_{{v_j}{v_i}}}} = 0 \quad {v_i} \notin S,\ {v_i} \notin D,\ \left\langle {{v_i}{v_j}} \right\rangle \in \end{aligned}$$

The capacity constraints of links are formulated as follows:

4 $$\begin{aligned} 0 \le {f_{{v_i}{v_j}}} \le {C_{{v_i}{v_j}}},\ \left\langle {{v_i}{v_j}} \right\rangle \in E \end{aligned}$$

For the network with *N* source and *N* destination nodes, respectively, there exist $K = N \times N$ origin-destination (OD) pairs. The multi-source and multi-destination maximum flow model can be described as:

5 $$\begin{aligned} Max{\hspace{1.0pt}} {\hspace{1.0pt}} {\hspace{1.0pt}} {\hspace{1.0pt}} {\hspace{1.0pt}} \sum \limits _{k = 1}^K {\sum \limits _{p \in {P_k}} {w_{kp} } } \end{aligned}$$

where $S_k$ and $D_k$ are the source node and destination node of OD pair *k*, respectively; $P_k$ is its path set; $w_{ke}$ denotes the traffic of path set $P_k$ traversing link *e*. $w_{kp}$ should follow:

6 $$\begin{aligned}&w_{kp} \ge 0, \quad \forall k = 1,2,\ldots ,K,\ \forall p \in {P_k} \end{aligned}$$

7 $$\begin{aligned}&w_{ke} \le {C_e}, \quad \forall k = 1,2, \cdots K,\ \forall e \in E \end{aligned}$$

8 $$\begin{aligned}&\sum \limits _{k = 1}^K {w_{ke} } \le {C_e}, \quad \forall e \in E \end{aligned}$$

9 $$\begin{aligned}&\sum \limits _{p \in {P_k}} {w_{kp}} = {\Gamma ^k},\quad \forall k = 1,2, \cdots K \end{aligned}$$

*Minimum Network Energy Consumption:* To realize minimum network energy consumption, we define a rate-adaptive energy consumption function as:

10 $$\begin{aligned} F({x_{ij}}) = {\left\{ \begin{array}{ll} 0, & {x_{ij}} = 0 \\ \beta \cdot \left[ {\delta \cdot {C_{ij}} + (1 - \delta ) \cdot \frac{{{x_{ij}}^2}}{{{C_{ij}}}}} \right] , & 0 < {\hspace{1.0pt}} {x_{ij}} \le {C_{ij}} \end{array}\right. } \end{aligned}$$

where $x_{ij}$ and $C_{ij}$ are the load and capacity of link $l_{ij}$; $\delta$ and $\beta$ are function curve coefficients. When link $l_{ij}$ is closed, its load and energy consumption are zero; or otherwise their relationship is described by Equation 10. The minimum network energy consumption is to minimize the total energy consumption $\sum \limits _{(i,j) \in E} {F({x_{ij}})}$ of networks.

**Algorithm 2:**
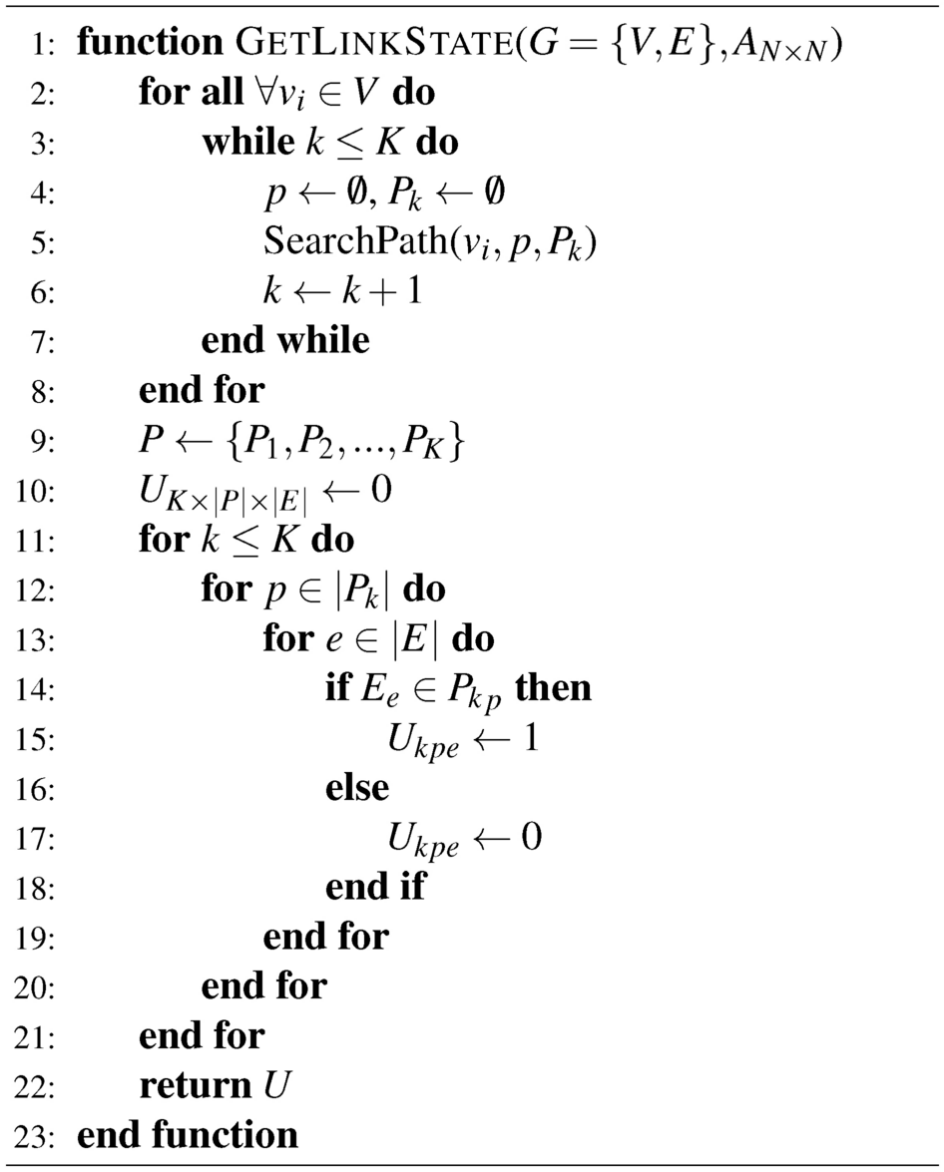
Link State Calculation Algorithm

*Max–min Model:* To achieve maximum energy efficiency, the maximum throughput and minimum energy consumption of networks are aimed at. Our max–min model is written as:

11 $$\begin{aligned} Max \quad \sum \limits _{k = 1}^K {\sum \limits _{p \in {P_k}} {w_{kp} } } - \lambda \sum \limits _{(i,j) \in E}{F({x_{ij}})} \end{aligned}$$

where link utilization is $w_{kp} \ge 0{\hspace{1.0pt}} {\hspace{1.0pt}}$, $\forall k = 1,2,\ldots ,K{\hspace{1.0pt}} {\hspace{1.0pt}}$, $\forall p \in {P_k}$; link capacity constraints are defined as:

12 $$\begin{aligned}&\sum \limits _{k = 1}^K {\sum \limits _{p \in P_k^H} {{U_{kpe}}w_{kp} } \le \alpha \times {C_e}}, \quad \forall e \in E \end{aligned}$$

13 $$\begin{aligned}&\sum \limits _{p \in {P_k}} {w_{kp} \le } {\Gamma ^k}, \quad \forall k = 1,2,\ldots ,K \end{aligned}$$

14 $$\begin{aligned}&\sum \limits _{p \in P_k^H} {{U_{kpe}}w_{kp} } \le \alpha \times {C_e}, \quad \forall k = 1,2, \cdots K, \forall e \in E \end{aligned}$$

The upper constraint of path traffic is:

15 $$\begin{aligned} \sum \limits _{p \in {P_k}} {w_{kp} \ge \beta } {\Gamma ^k}, \quad \forall k = 1,2,\ldots ,K \end{aligned}$$

The maximum hop number constraint is:

16 $$\begin{aligned} hop(p) \le H,\quad \forall p \in {P_k}, \forall k = 1,2,\ldots ,K \end{aligned}$$

where $\lambda$is a Lagrange parameter. The optimization model is an integer linear programming model, which can be solved exactly by CPLEX^21^.

*Optimal Solution:* First, we propose a the link state matrix calculation algorithm, shown as Algorithm 1 and 2. For each OD pair with its source node $S_k$ and the destination node $D_k$, initiate ${P_k} = \emptyset$. Initialize the path set of data plane as $P = \emptyset$. Then calculate *P* according to the depth-first search algorithm finding out all paths set between any two nodes in the directed connected graph. For $\forall {v_i} \in V$ and $\forall \left\langle {{v_i}{v_j}} \right\rangle \in E$, perform depth-first search, where “1” represents the node has been visited; “0” represents the node is not visited. Calculate associated edge set $RE_i$ of node $v_i$ according to adjacency matrix. If edge $\left\langle {{v_i}{v_j}} \right\rangle \in R{E_i}$ and the mark of $\left\langle {{v_i}{v_j}} \right\rangle$ and ${v_j}$ is “0”, then mark both ${v_j}$ and $\left\langle {{v_i}{v_j}} \right\rangle$ as “1”. If ${v_j} \ne {D_k}$, set ${v_i} = {v_j}$ and continue the process above to conduct deep search from node ${v_j}$ until ${v_j} = {D_k}$ and save *p* as successful path to $P_k$. Otherwise, abandon this path and keep the mark values of the vertex and links. Back to node $v_i$, if ${v_i} \ne {S_k}$, continue the process above to obtain the path set of the entire data plane $P = \left\{ {{P_1},{P_2}, \cdots ,{P_k}} \right\}$. Then calculate link state matrix. Initiate link set *E* of the data plane and path set $P_k^H$ of combinations *k* for $\forall p \in {P_k}^H$ and $\forall e \in E$ . If the path *p* occupies link *e* , let ${U_{kpe}} = 1$ , or otherwise ${U_{kpe}} = 0$. Given adjacency matrix ${A_{N \times N}}$, traffic request matrix ${\Gamma _{N \times N}}$, link capacity ${C_e}$, maximum link utilization ratio $\alpha$, lower bound factor of end-to-end traffic rate $\beta$ and the obtained *U*, build the model in 10 and solve it using CPLEX to attain energy-efficient data plane and control plane.

**Fig. 3:**
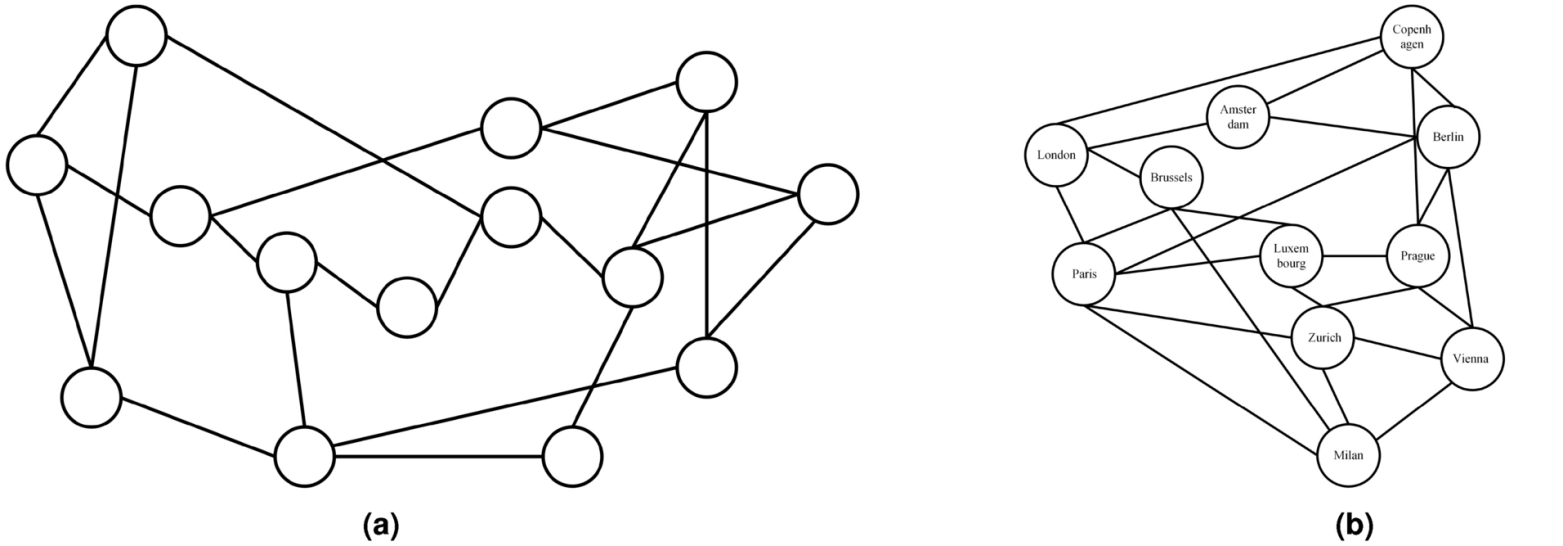
Physical topologies of the networks for simulation. (**a**) Physical topology of the NSF network. (**b**) Physical topology of the COST239 network.

## Numerical results and discussions

In this section we evaluate the performance of the proposed dynamic max–min planning approach for energy-efficient networking. NSF^22^and COST239^23^network topologies are used for our simulation topology, which are commonly used in evaluating the performance of 5G transport networks and carrier networks^22^. The physical topologies of the two networks are shown in Fig. 3. We implement the proposed system model in Fig. 2 with SDN tool chain, in an Ubuntu 20.04 environment. For data plane, we employ Mininet^24^for creating the realistic virtual network, where Open vSwitch and hosts are running and communicating with each other. Every host in the network is simulated using the Linux network namespaces with its own individual processes with separate network interfaces, routing tables, and ARP tables. The links between hosts and switches are simulated by virtual ethernet (veth) pairs. We generate the traffic of the TCP flows between each source and destination hosts using iperf. For control plane, we employ ONOS controller^25^, which provides capabilities of distributed controllers to realize the multi-controller structure in Fig. 2. We use Docker to deploy multiple ONOS instances and collaborate with each other to provide better services than a single controller. Multiple ONOS instances forms a cluster, where each node is responsible for the state of a subsection of the data plane. The state information of the subsection is spread within the cluster by the corresponding node as an event. The event is generated in the store and shared within cluster via the services deployed in the distributed stores^25^. The northbound API between control and data plane is integrated in ONOS, which is responsible for providing the overview of network state and distributing the control instructions, including Topology, Path Selection, Flow Manager, etc. Specifically, the decision-making module in Fig. 2 interacts with the controllers in control plane using northbound API provided by subsystems of ONOS, namely device subsystem, link subsystem, topology subsystem, flow-rule subsystem, etc. The southbound API between control plane and data plane is OpenFlow. Numerical experiments are conducted in CPLEX to calculate the optimal planning results.

**Fig. 4:**
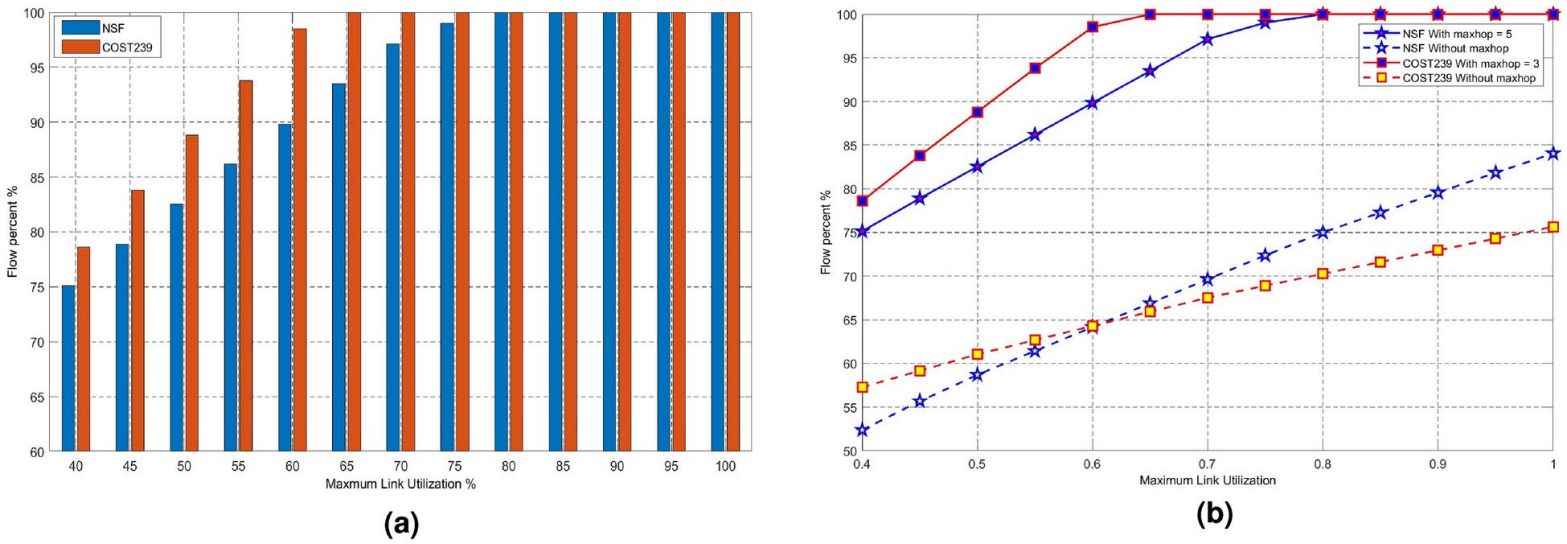
Performance analysis for transmitted Flows with maximum link utilization. (**a**) Percentage of transmitted flows with maximum link utilization, (**b**) Change of transmitted Flows with maximum link utilization.

Figure 4(a) shows the percentage of transmitted flows with maximum link utilization when the network link utilization is $0.4 \le \alpha \le 1.0$, the traffic allowed through link increases with the link utilization. In order to verify the performances of our algorithm, the simulations analyze the impact of different parameters on the data plane of the network, compared with the classical max–min Fairness (MMF) algorithm, which is represented as “Without MaxHop” in the results^26^. MMF is a resource allocation algorithm that satisfies as much traffic demand as possible while balancing fairness. It is one of the commonly used algorithms in network energy efficiency planning problems. The advantage of algorithms lies in their high average throughput and resource utilization, as well as their high stability, making it less likely for a single task to suddenly affect other tasks. However, the disadvantage of MMF is that it is generally only applicable to a single shared resource. For our algorithm and MMF algorithm, with the changes of link utilization, diagram of the percentage change of network traffic accounting for the demand of end-to-end traffic is shown in Fig. 4b. Figure 4b indicates the change of transmitted Flows with maximum link utilization. Our algorithm is simulated on the topologies of NSF and COST239, respectively, compared with MMF algorithm. From Fig. 4b, we can see that at the same maximum link utilization, flow transmission percentage of our algorithm is much bigger than that of the MMF algorithm. Hence, given the same maximum link utilization, that is, when energy consumption is the same, the proposed algorithm can carry more flows, which means higher energy efficiency. Meanwhile, with the increasing of link maximum utilization, the flow value allowed through the link increases and traffic transmission value increases in the data plane network.

**Fig. 5:**
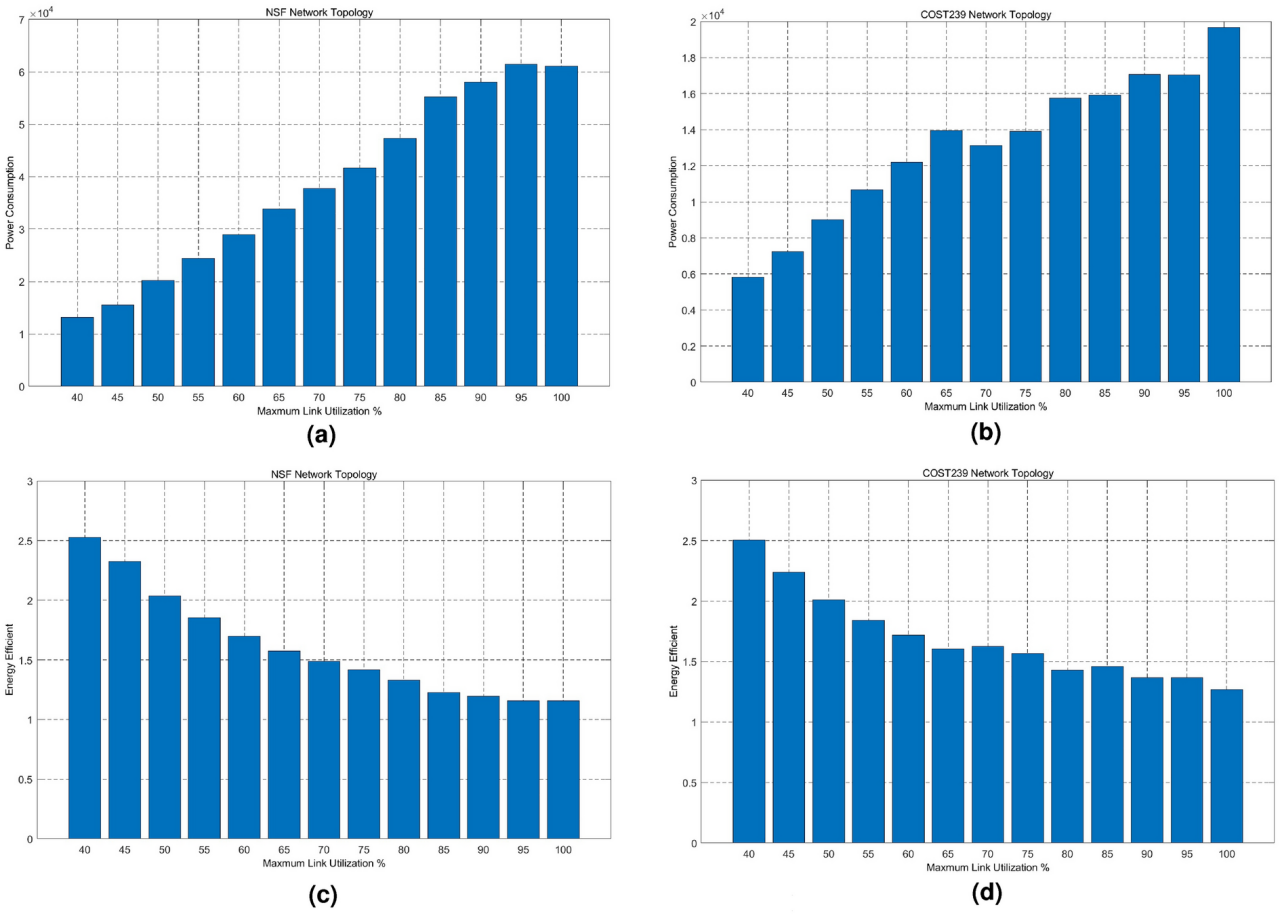
Impact of maximum link utilization on network power consumption and energy efficiency. (**a**) Network power consumption for NSF, (**b**) Network power consumption for COST239, (**c**) Network energy efficiency for NSF, (**d**) Network energy efficiency for COST23.

**Fig. 6:**
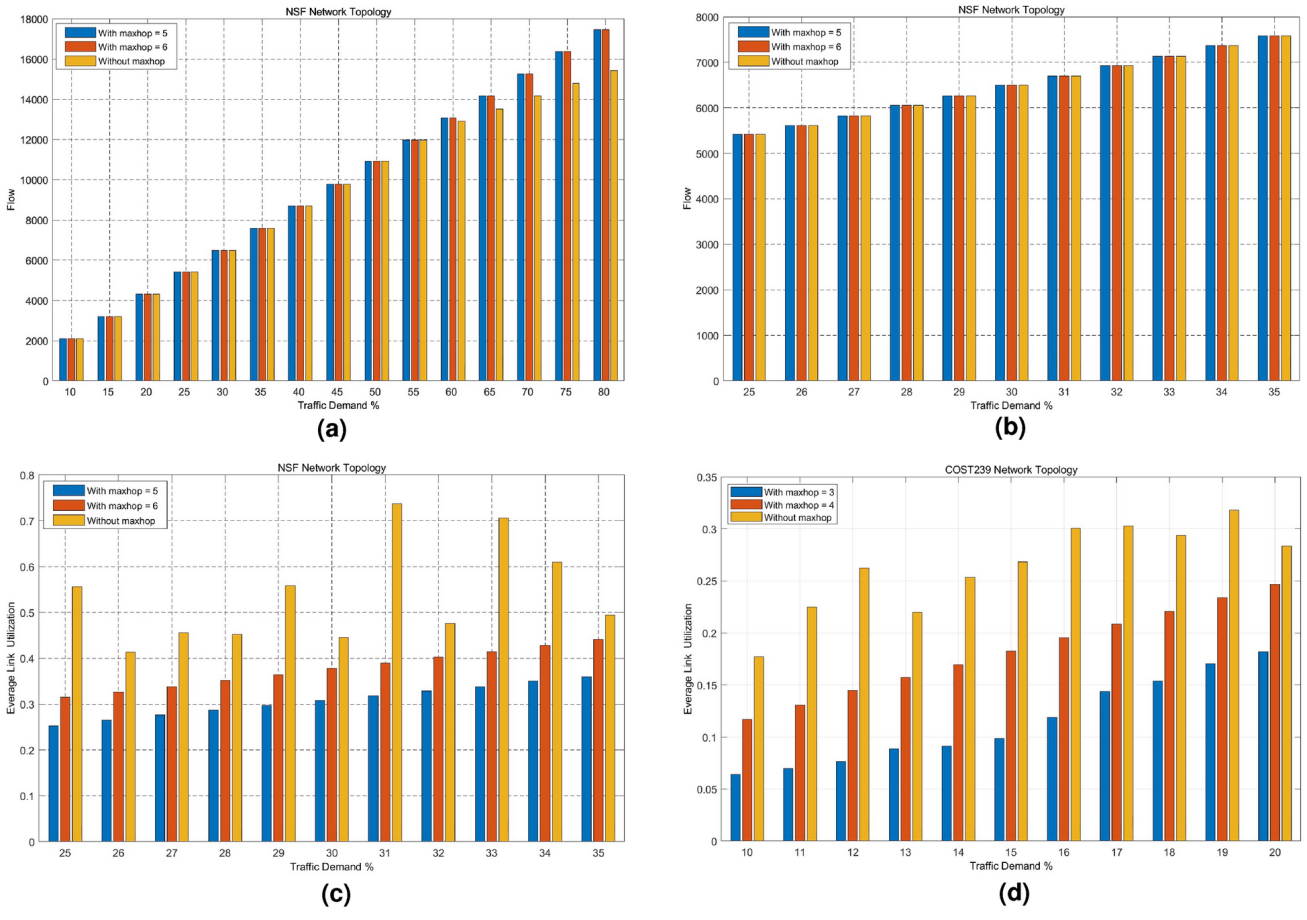
Impact of different hops on transmitted flows and average link utilization. (**a**) Change of transmitted flows for NSF, (**b**) Change of transmitted flows for COST239, (**c**) Average link utilization for NSF, (**d**) Average link utilization for COST239.

Form Fig. 5a, b, it is easy to find that network energy consumption increases with maximum link utilization. Figure 5c, and d indicate the energy efficiency of our approach.

Figure 6a shows that when the real traffic request is bigger than 60%, the forwarding traffic of MMF algorithm is smaller than that of our algorithm. In order to make fair comparison for both algorithms, we choose $0.25 \le \varepsilon \le 0.35$ to analyze their performance. Figure 6b indicates the traffic transmission of MMF and our algorithm at $0.25 \le \varepsilon \le 0.35$. As seen in Fig. 6(a) and (b), MMF and our algorithm have the same traffic transmission values. Figure 6c shows the average link utilization variation diagram of NSF in data plane network with different traffic requests for our algorithm and MMF algorithm. The maximum link utilization rate is $\alpha = 0.9$ and the real traffic request is $0.25 \le \varepsilon \le 0.35$. As can be seen from Fig. 6c, the link average utilization of MMF (without maxhop) is bigger than those of the proposed algorithm (with maxhop) at the same traffic request. This is because the probability of each link occupied increases with the increasing of the number of hops. The smaller the number of hops is, the smaller the path goes through the links, and the lower the link utilization is. With the increasing of traffic request, the average link utilization of our algorithm increases, but that of MMF changes irregularly. Figure 6d illustrates the average link utilization of COST239 in data plane network with different traffic requests for both algorithms.

**Fig. 7:**
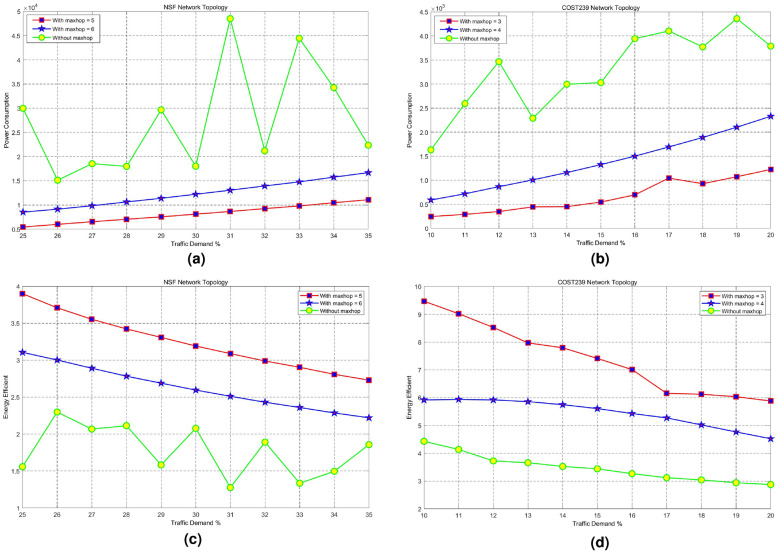
Impact of different hops on energy consumption and energy efficiency. (**a**) Energy consumption for NSF, (**b**) Energy consumption for COST239, (**c**) Energy efficiency for NSF, (**d**) Energy efficiency for COST239.

Figure 7a shows the link energy consumption of NSF in data plane network with different traffic requests for both algorithms. The maximum link utilization rate is $\alpha = 0.9$ and the real traffic request rate is $0.25 \le \varepsilon \le 0.35$. As can be seen from Fig. 7a, the energy consumption of our algorithm is lower than that of MMF with the same traffic transmission. With the increasing of traffic request, traffic values through links increase and the energy consumption of network links is proportional to the square of link flow. As a result, data plane link energy consumption of our algorithm increases. Figure 7a shows that the link utilization of MMF algorithm does not increase with the increasing of link utilization. Consequently, link energy consumption changes irregularly, which is caused by the dynamic traffic patterns of the end-to-end traffic flows. The fairness calculation of the MMF algorithm is based on a snapshot of traffic demands as a given moment. The dynamic changes in traffic patterns over time may cause the MMF algorithm to readjust resource allocations. These adjustments can lead to temporary deviations from the optimal fairness distribution, introducing randomness in resource allocation. The dynamic changes are represented as the changes of maximum link utilization rate and the real traffic request rate in this paper. Figure 7b indicates the link energy consumption of COST239 in data plane network with different traffic requests for both algorithms. The maximum link utilization is $\alpha = 0.8$ and the real traffic request is $0.1 \le \varepsilon \le 0.2$. Figure 7b shows that the energy consumption of our algorithm is lower than that of MMF with the same traffic transmission. Data plane link energy consumption of our algorithm increases with the increasing of traffic request. Figure 7c plots the energy efficiency variation diagram of NSF in data plane network with different traffic requests for both algorithms. The maximum link utilization is $\alpha = 0.9$ and the real traffic request is $0.25 \le \varepsilon \le 0.35$ . Figure 7c demonstrates that the energy efficiency of our algorithm is higher than that of MMF with the same traffic transmission. Network efficiency is defined as the transmitting flow value with one unit energy consumption. The bigger the value, i.e., the energy consumption of transmitting one unit flow is smaller, the better the performances. Link utilization and link energy consumption increase with the increasing of traffic request. Consequently, network energy efficiency decreases with the increasing of link loads. Figure 7d plots the energy efficiency variation diagram of COST239 in data plane network with different traffic requests for both algorithms. The maximum link utilization is $\alpha = 0.8$ and the real traffic request is $0.1 \le \varepsilon \le 0.2$ . Figure 7c indicate that network energy efficiency of our algorithm is higher than those of MMF and energy efficiency decreases with the increasing of network traffic requests.

## Conclusions and future research

In this paper we have rethought the max–min planning framework on energy efficiency for intelligent networking of 5G networks. The proposed approach takes into account combining network connectivity, maximum network flow, and minimum energy consumption. Our framework uses new max–min planning to attain high energy efficiency and network performance according to the dynamic end-to-end traffic demands. Our future research will focus on modeling the impact of burst traffic on energy efficiency and quality of service. Finally, future work will aim to balance network-wide links for improved energy efficiency of intelligent networking in 5G networks.

## Data Availability

All data generated or analyzed during this study are included in this published article.
